# Discrimination, social support, and mental health in reproductive-aged women: the All of Us research program

**DOI:** 10.1186/s12905-026-04353-x

**Published:** 2026-03-18

**Authors:** Suheilah Abdalla, Aize Cao, Judith Dike, Sanghyuk S. Shin, Candice Taylor Lucas, Yuqing Guo

**Affiliations:** 1 Sue & Bill School of Nursing, University of California, Irvine, Irvine, CA USA; 2 Department of Biomedical Data Science, School of Applied Computational Sciences, Meharry Medical College, Nashville, TN USA; 3 Department of Pediatrics, School of Medicine, University of California, Irvine, CA USA

**Keywords:** Depression, Anxiety, Discrimination, Social Support, Reproductive-Aged Women

## Abstract

**Background:**

Discrimination and social isolation during the coronavirus disease (COVID-19) pandemic exacerbated mental health challenges in the United States (U.S.). Such exposures may have compounded effects for reproductive-aged women who are at increased risk of adverse mental health outcomes due to their unique psychosocial vulnerabilities. The purpose was to describe the prevalence of depression and anxiety symptoms in reproductive-aged women during the pandemic, and examine associations between discrimination, social support, and depression/anxiety symptoms.

**Methods:**

Data were retrieved from the National Institutes of Health (NIH) All of Us Research program. Of the 14,250 participants who met the inclusion criteria—completion of the first COPE survey in May, June, or July 2020, assigned female sex at birth, and age 18–50 years—12,904 remained for final analysis after excluding those with missing data. The Everyday Discrimination Scale measured experiences of discrimination. Social support was evaluated using the Medical Outcomes Study Social Support Scale. The Patient Health Questionnaire-9 (PHQ-9) and Generalized Anxiety Disorder-7 (GAD-7) assessed depression (mean ± SD = 7.04 ± 5.67) and anxiety symptoms (mean ± SD = 7.14 ± 5.30). Descriptive statistics, linear regression, and mediation analyses were conducted.

**Results:**

Approximately one-third of reproductive-aged women reported moderate-to-severe depression or anxiety symptoms. Women identifying as Other or Mixed race/ethnicity exhibited the highest rates. Higher discrimination was significantly associated with greater depression (b = 0.45, *p* < 0.001) and anxiety symptoms (b = 0.40, *p* < 0.001). Social support mediated the association between discrimination and depression symptoms (mediation effect: b = 0.10, *p* < 0.001) and between discrimination and anxiety symptoms (mediation effect: b = 0.06, *p* < 0.001).

**Conclusions:**

This study revealed the interplay between discrimination, social support, and mental health. Women experiencing discrimination tended to perceive less available social support, which was associated with higher depression/anxiety symptoms. These results provide preliminary evidence that social support may serve as a key strategy to counteract the detrimental effects of discrimination on mental health for reproductive-aged women in the U.S. These findings emphasize the need for holistic mental health care incorporating comprehensive assessments and personalized interventions to effectively manage mental health symptoms and address the social determinants of health.

## Background

Coronavirus disease (COVID-19) has had a significant impact on both physical and mental health in the United States (U.S.) [1]. Although various measures were implemented to curb the virus’s spread, such as closing businesses and schools and changing healthcare policies [2], mass deaths in 2020 led to widespread emotional distress [3]. The pandemic disproportionately impacted racialized communities, resulting in higher death rates among these groups [4]. These factors played a critical role in mental health, particularly for reproductive-aged women, as they are at increased risk of navigating multifactorial challenges such as limited access to childcare, gender disparities, and/or intimate partner violence [5–8].

Discrimination, both racial and non-racial, is a psychosocial stressor that unfairly treats individuals, stigmatizing and separating social groups from one another [9, 10]. Studies show that such mistreatment is associated with a wide range of mental health issues, such as depression and anxiety [11, 12]. The COVID-19 pandemic exacerbated the impact of discrimination in the U.S. [13, 14]. A recent study using the All of Us Research Program (AoURP) revealed that both non-racial and racial discrimination were associated with increased depression in the U.S. general population, including both women and men aged 18 years or older [15]. Similarly, in another nationally representative sample, individuals identifying as Asian/Asian American or Pacific Islander or African American/Black had the highest reported levels of discrimination during the COVID-19 pandemic and experienced high levels of emotional distress among women and men aged 18 years or older [16].

Beyond the well-documented negative impact of discrimination on mental health, research demonstrates that inadequate social support is also linked to depressive symptoms [17, 18]. During the COVID-19 pandemic, decreased social support due to mandated social isolation measures was associated with increased depression/anxiety among individuals aged ≥ 18 years [16, 19]. The Social Support Deterioration Model (SSDM) posits that traumatic or stigmatizing events (e.g., discrimination) can reduce both perceived and actual social support, subsequently leading to increased mental health challenges [20]. Supporting this framework, one study of African American college students found that decreased perceived social support partially mediated the relationship between racial discrimination and depression [21].

Existing literature establishes discrimination as a risk factor for mental health problems [22, 23], while social support serves as a protective factor of emotional well-being [11, 22]. Research indicates that individuals who experience discrimination report increased insecurity in social interactions which is associated with social isolation [24]. Notably, a robust body of research demonstrates the beneficial role of social support as a protective factor for mental health, as well as a buffer for the effects of discrimination on mental health symptoms [11, 22, 23, 25, 26]. Relatively few studies examine how social support mediates the pathways between discrimination and poorer mental health [27, 28].

Growing evidence indicates gender-specific differences for mental health, showing that women experience higher rates of depression and/or anxiety than men [29, 30]. However, women remain underrepresented in gender-specific health research, particularly racial and ethnically diverse women, contributing to disparities in mental health knowledge and interventions [31, 32]. Studies focusing on mental health in reproductive-aged women are scarce, although this population faces unique stressors affecting their fertility, gynecologic health, and overall well-being [33]. From a life-course perspective, optimizing their health is crucial as their well-being has implications not only for their own lives but also for current and future generations [32].

There is limited research examining the relationships between discrimination, social support, and mental health problems – particularly both depression and anxiety – in reproductive-aged women in the U.S. To address this gap, we utilized the large, diverse population enrolled in the National Institutes of Health (NIH) All of Us Research Program (AoURP) with three aims: (1) to describe the prevalence of depression and anxiety in women aged 18 to 50 years old; (2) to examine the associations between discrimination and depression/anxiety; and (3) to explore social support as a potential mediator for the associations of discrimination and depression/anxiety in this population.

## Methods

### Study design

This study used data from AoURP, a NIH initiative with the goals of engaging at least one million diverse participants across the U.S. The program prioritizes accessibility and inclusivity for populations underrepresented in healthcare research, including racial and ethnic minorities and individuals from varied age groups, geographic regions, and socioeconomic backgrounds. Data are collected through health questionnaires, electronic health records (EHRs), physical measurements, digital health technology, and biospecimens. Beginning in May 2018, the program has enrolled participants aged 18 and older through a network of over 300 approved sites [34].

### Participants

Institutional Review Board approval was obtained through the NIH AoURP. Participants provided informed consent before enrollment. Data was de-identified before being made available to researchers through the All of Us Researcher Workbench, a secure cloud environment. We used AoURP version 8, an ongoing cohort of 413,360 participants [34]. Our study extracted data from the COVID-19 Participant Experience (COPE) survey, a component of AoURP designed to assess the pandemic’s effects on participants’ lives and health. Inclusion criteria were (1) completion of the first COPE survey in May, June, or July 2020, (2) assigned female sex at birth, and (3) being aged 18–50. Of the 61,959 participants who completed the first COPE survey in May, June, or July 2020, 14,250 women aged 18–50 were identified for this study [35, 36].

### Measures

The COPE survey comprises validated questionnaires that measure COVID-19 symptoms, physical and emotional health, coping strategies, and social distancing behaviors. For this cross-sectional analysis of the COPE survey, we used data from participants who completed the survey between May and July 2020 and focused on four questionnaires assessing discrimination, social support, depression symptoms, and anxiety symptoms, along with relevant demographic information.

#### Discrimination

The Everyday Discrimination Scale is a 9-item questionnaire that measures participants’ self-perceived experiences of discrimination (racial and non-racial) due to various reasons such as gender, race, age, religion, weight, height, sexual orientation, education, or income level during the past month (e.g., “How often did you receive poorer service than other people?” and “How often did people act as if they were better than you were?”). Responses are rated using a 4-point Likert scale (0 = never, 1 = a few times a month, 2 = at least once a week, and 3 = almost every day). The total score, calculated by summing responses to all 9 items, ranges from 0 to 27, with higher scores indicating greater frequency of self-perceived discrimination [37]. The scale demonstrated acceptable reliability in this sample (Cronbach’s α = 0.71).

#### Social support

Perceived social support was measured using 10 items from the RAND Medical Outcome Study Social Support Survey. This survey assesses four types of support experienced in the past month, including tangible, emotional, informational, and positive social interaction support. Example questions include “How often can you find someone to have a good time with?” and “How often can you find someone to understand your problems?” Responses are rated on a 5-point Likert scale (1 = none of the time, 2 = a little of the time, 3 = some of the time, 4 = most of the time, 5 = all of the time). The total score, calculated by summing responses to all 10 items, ranges from 10 to 50, with higher scores indicating greater perceived social support [38]. The scale demonstrated excellent reliability in this sample (Cronbach’s α = 0.94).

#### Depression symptoms

Depressive symptoms were assessed using the Patient Health Questionnaire (PHQ-9), a 9-item screening tool that evaluates the DSM-IV criteria for depression. Participants reported their experiences over the past two weeks on a 4-point Likert scale (0 = not at all, 1 = several days, 2 = more than half the days, 3 = nearly every day). Example questions are “How often have you been bothered by feeling down, depressed or hopeless” and “How often have you been bothered by trouble falling, or staying asleep or sleeping too much?” A total score, calculated by summing responses to all 9 items, ranges from 0 to 27, with a cut-off score of 10 or above indicating moderate-to-severe depressive symptoms [39]. The scale demonstrated good reliability in this sample (Cronbach’s α = 0.86).

#### Anxiety symptoms

Anxiety symptoms were measured using the Generalized Anxiety Disorder-7 (GAD-7), a 7-item questionnaire screening for generalized anxiety symptoms experienced in the past two weeks. Participants responded on a 4-point Likert scale (0 = not at all, 1 = several days, 2 = more than half the days, 3 = nearly every day). Example questions are “How often have you been bothered by not being able to stop or control worrying?” and “How often have you been bothered by becoming easily annoyed or irritated?” A total score, calculated by summing responses to all 7 items, ranges from 0 to 21, with a cut-off score of 10 or above indicating moderate-to-severe anxiety symptoms [40]. The scale demonstrated excellent reliability in this sample (Cronbach’s α = 0.91).

#### Demographics

Demographics were obtained from surveys administered by the AoURP and the EHRs, respectively. In the AoURP, race and ethnicity were separated into two distinct questions, allowing participants to select the racial and ethnic categories they identified with most closely. The AoURP provided participants with the option to choose Hispanic or non-Hispanic ethnicity. Participants then self-identified their race as Asian, Black/African American, White, Other single population (i.e., “None of these”, “Native Hawaiian or Other Pacific Islander”, “None Indicated”), or More than one population. In our study, we recoded race and ethnicity into a single variable with categories: non-Hispanic (NH)-White, NH-Black/African American, NH-Asian, Hispanic (any race), Other, and Mixed (more than one race). Additional categorical variables included education, employment, annual household income, marital status, health insurance status, homeownership, and the history of previous mood disorder diagnoses (extracted from the EHRs). Age was treated as a continuous variable (see Table 1).

**Table 1:** Descriptives of Key Variables (*N* = 12,904)

Variable	Participants *n* (%)
Categorical variables	
Race/Ethnicity	
NH-White	9,404 (72.88)
Hispanic	1,500 (11.62)
NH-Black/African American	977 (7.57)
NH-Asian	564 (4.37)
Mixed	374 (2.90)
Other	85 (0.66)
Education	
Bachelor’s degree or higher	9,316 (72.19)
Some college	2,694 (20.88)
High school/GED	733 (5.68)
Did not complete high school	161 (1.25)
Employment	
Employed	9,801 (75.95)
Not currently employed	3,103 (24.05)
Annual Income $	
< 10,000	816 (6.32)
10,000–50,000	3,557 (27.57)
50,000–100,000	3,864 (29.94)
100,000–150,000	2,261 (17.52)
150,000–200,000	1,137 (8.81)
> 200,000	1,269 (9.83)
Married/living with a partner	7,497 (58.10)
Had health insurance	12,537 (97.16)
Owned a home	6,165 (47.78)
History of mood disorder diagnoses	2,855 (22.11)
Continuous variables	
Age	36.93 (8.03)
Discrimination	3.15 (3.60)
Social support	39.39 (9.75)
Depression symptoms	7.04 (5.67)
Anxiety symptoms	7.14 (5.30)

NH: non-Hispanic; Mixed: more than one race/ethnicity (NH-White, Hispanic, NH-Black/African American, or NH-Asian); GED: General Educational Development

### Data analysis

There were 14,250 participants who filled out the first-time COPE survey in May, June or July 2020. Observations with missing values for demographics, discrimination, depression/anxiety symptoms, and social support (*n* = 1,346) were excluded. The overall missingness rate across key variables was 9.4%, which is below the 10% threshold at which complete-case analysis is generally acceptable and imputation provides limited additional benefit [41, 42]. The final analysis data set included 12,904 participants.

#### Covariates

All demographic variables were used as covariates, as they were identified as potential confounding factors of mental health outcomes in a previous study using the AoURP [15]. Notably, race/ethnicity was included as a category in our study as a proxy for systematic embodied effects of racism, rather than biological or genetic differences [43].

#### Statistical analysis

Analyses were performed on the AoU Researcher Workbench using R software version 4.2.2, with p-values < 0.05 considered statistically significant. Three main statistical analyses were conducted: (1) descriptive statistics were used to report the prevalence of moderate-to-severe depression and anxiety across race/ethnicity categories. Depression and anxiety were operationalized as binary variables using established cut-off scores: participants with PHQ-9 total scores ≥ 10 were classified as having moderate-to-severe depressive symptoms (hereafter “depression”), and those with GAD-7 total scores ≥ 10 were classified as having moderate-to-severe anxiety symptoms (hereafter “anxiety”); chi-square tests were conducted to compare prevalence across race/ethnicity groups, followed by post hoc pairwise comparisons with Bonferroni correction to identify specific between-group differences; (2) linear regression analyses to investigate associations between discrimination and depression/anxiety symptoms, using continuous PHQ-9 and GAD-7 total scores as dependent variables, while controlling for the covariates; and (3) mediation analysis to explore the mediating effects of social support on the associations between discrimination and depression/anxiety symptoms (continuous PHQ-9 and GAD-7 total scores as dependent variables), while controlling for covariates. The mediation package in R was used, with bootstrapping (1000 replicates) to estimate standard errors and p-values for the total, direct, and mediated effects [44]. In addition, subgroup analyses were employed for NH-White, NH-Black/African American, NH-Asian, and Hispanic women, and Mixed/Other.

## Results

### Demographics of participants

Participants (36.93 ± 8.03 years) were predominantly NH-White (72.88%), highly educated with bachelor’s degrees or higher (72.19%), employed (75.95%), and earning $50,000/year or higher (66.10%). Approximately half (47.78%) owned homes, and 58.10% were married or cohabiting. Nearly one-quarter (22.11%) reported a history of mood disorder diagnosis (see Table 1 for detailed demographic data).

### Prevalence of depression and anxiety symptoms

Overall, 28.21% of participants reported moderate-to-severe depression. Depression prevalence varied significantly by race/ethnicity (χ² (5) = 17.26, *p* = 0.004). Pairwise analyses showed that the participants in the Other category had significantly higher depression rates than NH-White (χ² = 5.17, *p* = 0.02), Hispanic (χ² = 4.71, *p* = 0.03), NH-Black/African American (χ² = 6.46, *p* = 0.01), and NH-Asian participants (χ² = 8 0.37, *p* = 0.004). Similarly, those identifying as the Mixed category had significantly higher depression rates than NH-White (χ² = 5.49, *p* = 0.02), Hispanic (χ² = 4.18, *p* = 0.04), NH-Black/African American (χ² = 6.99, *p* = 0.008), and NH-Asian participants (χ² = 9.53, *p* = 0.002). These findings indicate that depression prevalence is not uniform across race/ethnicity groups, with the greatest burden among those identifying as Other (40.00%) or Mixed (33.96%), followed by Hispanic (28.40%), NH-White (28.24%), NH-Black/African American (26.51%), and NH-Asian participants (24.47%).

The overall prevalence of moderate-to-severe anxiety was 28.22%. As shown in Table 2, there were significant differences in anxiety prevalence across race/ethnicity groups (χ² (5) = 1177.65, *p* < 0.001). Pairwise analyses revealed that participants in the Other category had significantly higher anxiety rates than Asian participants (χ² = 6.98, *p* = 0.008). Comparisons between the Other and NH-White (χ² = 3.17, *p* = 0.08), Hispanic (χ² = 2.82, *p* = 0.093), and NH-Black/African American participants (χ² = 3.79, *p* = 0.05) showed marginally significant differences. Additionally, individuals identifying as the Mixed category had significantly higher anxiety rates compared to Asian participants (χ² = 4.40, *p* = 0.04). However, comparisons between those identifying as Mixed and NH-White (χ² = 0.40, *p* = 0.53), Hispanic (χ² = 0.23, *p* = 0.63), and NH-Black/African American participants (χ² = 0.94, *p* = 0.33) were not significant. These findings indicate that anxiety prevalence is not uniform across race/ethnicity groups, with the highest burden observed among individuals identifying as Other (36.47%) or Mixed (30.75%), and NH-Asian participants had the lowest anxiety prevalence (22.87%).

**Table 2:** Prevalence of Depression and Anxiety Symptoms (*N* = 12,904)

Race/Ethnicity	Depression %	Anxiety %
NH-White	28.24	28.42
Hispanic	28.40	28.47
NH-Black/African American	26.51	27.33
NH-Asian	24.47	22.87
Mixed	33.96	30.75
Other	40.00	36.47

Depression: PHQ-9 total scores ≥ 10 indicate moderate-to-severe depressive symptoms; Anxiety: GAD-7 total scores ≥ 10 indicate moderate-to-severe anxiety symptoms; NH: non-Hispanic; Mixed: more than one race/ethnicity (NH-White, Hispanic, NH-Black/African American, or NH-Asian)

### Associations between discrimination and depression/anxiety symptoms

Table 3 shows the associations between discrimination and depression/anxiety symptoms using linear regression. We found that higher discrimination was significantly associated with greater depression (b = 0.45, *p* < 0.001) as well as with greater anxiety symptoms (b = 0.40, *p* < 0.001). Moreover, almost all covariates were found to be associated with depression and anxiety symptoms, except for health insurance.

**Table 3:** Linear Regression of Associations Between Discrimination and Depression/Anxiety Symptoms (*N* = 12,904)

Variable	Depression		Anxiety	
	b-estimate (SE)	*P* value	b-estimate (SE)	*P* value
Discrimination	0.45 (0.01)	< 0.001	0.40 (0.01)	< 0.001
Age	-0.04 (0.01)	< 0.001	-0.09 (0.01)	< 0.001
Race/Ethnicity				
NH-White	ref		ref	
Hispanic	-0.63 (0.15)	< 0.001	-0.46 (0.14)	0.001
NH-Black/African American	-1.79 (0.18)	< 0.001	-1.34 (0.17)	< 0.001
NH-Asian	-0.90 (0.23)	< 0.001	-1.21 (0.22)	< 0.001
Mixed	0.13 (0.27)	0.62	-0.17 (0.26)	0.53
Other	0.40 (0.48)	0.48	-0.01 (0.54)	0.98
Educational Level				
Bachelor’s degree or higher	ref		ref	
Less than high school/GED	-0.16 (0.43)	0.72	-0.27 (0.41)	0.52
High school/GED	0.05 (0.21)	0.82	-0.31 (0.20)	0.13
Some college	0.70 (0.12)	< 0.001	0.26(0.12)	0.03
Employment Status				
Employed	ref		ref	
Not currently employed	0.95 (0.12)	< 0.001	0.25 (0.11)	0.02
Annual Income				
100k – 150k	ref		ref	
< 10k	1.27 (0.25)	< 0.001	0.76 (0.24)	0.001
10k – 50k	0.90 (0.16)	< 0.001	0.39 (0.15)	0.01
50k – 100k	0.35 (0.14)	0.01	0.01 (0.13)	0.97
150k – 200k	-0.17 (0.19)	0.37	-0.20 (0.18)	0.26
> 200k	-0.25 (0.18)	0.17	-0.21 (0.17)	0.22
Marital Status				
Married/living with a partner	ref		ref	
Never married	0.20 (0.12)	0.09	-0.53 (0.11)	< 0.001
Divorced	0.65 (0.18)	< 0.001	-0.16 (0.18)	0.37
Separated	1.13 (0.37)	0.002	0.23 (0.35)	0.52
Widowed	-0.67 (0.30)	0.30	-0.61 (0.62)	0.32
Home Ownership				
Owned a home	ref			
Rent	0.51 (0.11)	< 0.001	0.49 (0.11)	< 0.001
Other Arrangments	1.18 (0.18)	< 0.001	0.87 (0.17)	< 0.001
History of mood disorder diagnoses				
No	ref			
Yes	1.94 (0.11)	< 0.001	1.29 (0.11)	< 0.001
Health Insurance				
No	ref			
Yes	0.01 (0.28)	0.96	-0.07 (0.27)	0.80

NH: non-Hispanic; Mixed: more than one race/ethnicity (NH-White, Hispanic, NH-Black/African American, or NH-Asian); GED General Educational Development

### Associations between discrimination, social support, and depression/anxiety symptoms

**Fig. 1:**
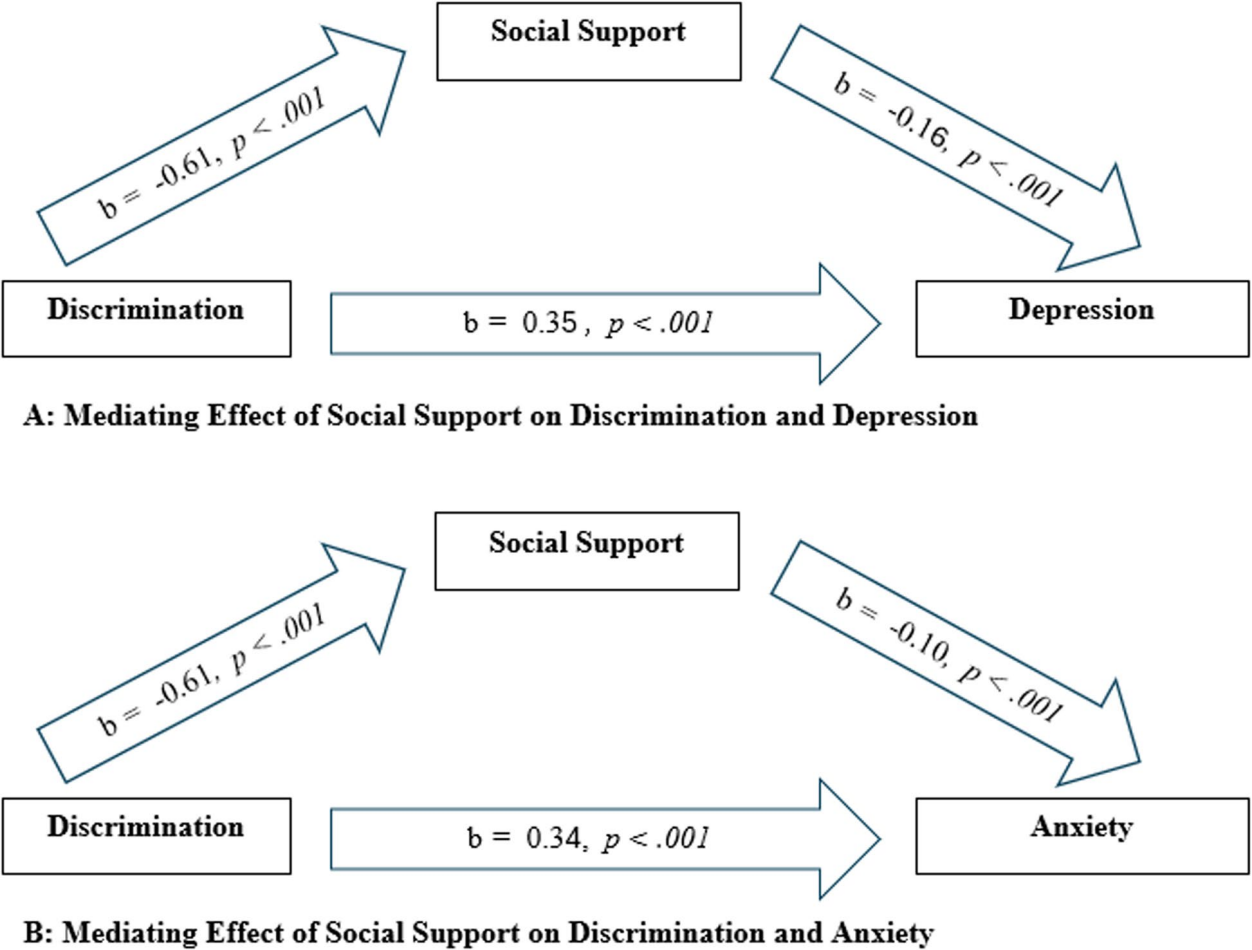
Social support as a mediator for associations between discrimination and depression/anxiety symptoms (*N*=12,904)

As illustrated in Fig. 1A and B, social support partially mediated the associations between discrimination and both depression and anxiety symptoms. Table 4 summarizes these mediated effects. For depression symptoms, the mediated effect was b = 0.10, *p* < 0.001; direct effect: b = 0.35, *p* < 0.001; and total effect: b = 0.45, *p* < 0.001. For anxiety symptoms, the mediated effect was b = 0.06, *p* < 0.001; direct effect: b = 0.34, *p* < 0.001; and total effect: b = 0.40, *p* < 0.001. Subgroup analyses revealed that social support mediated the associations between discrimination and depression/anxiety symptoms among NH-White, Hispanic, NH-Black/African American, and NH-Asian participants, but not among those identifying as Mixed/Other race/ethnicity.

**Table 4:** Summary of Mediated Effect of Social Support by Race/Ethnicity (*N* = 12,904)

Mental Health Symptoms	Total Effect	Direct Effect	Mediated Effect
Depression Symptoms			
All Participants	0.45***	0.35***	0.10***
Subgroup			
NH-White	0.46***	0.35***	0.11**
Hispanic	0.49***	0.40***	0.09***
NH-Black/African American	0.35***	0.32***	0.03***
NH-Asian	0.32***	0.26***	0.06***
Mixed/Other	0.41	0.25	0.16
Anxiety Symptoms			
All Participants	0.40***	0.34***	0.06***
Subgroup			
NH-White	0.40***	0.33***	0.07***
Hispanic	0.44***	0.39***	0.05***
NH- Black/African American	0.35***	0.32***	0.02***
NH-Asian	0.39***	0.35***	0.04***
Mixed/Other	0.33	0.22	0.11

NH: non-Hispanic; Mixed: more than one race/ethnicity (NH-White, Hispanic, NH-Black/African American, or NH-Asian); **p < .01; *** *p* < .0001

## Discussion

The study aimed to describe the prevalence of depression/anxiety symptoms and understand the relationships between discrimination, social support, and depression/anxiety in women between the ages of 18 and 50 during the early COVID-19 pandemic in the U.S. Nearly one-third of reproductive-aged women reported experiencing moderate-to-severe depression or anxiety symptoms early in the pandemic. Our findings are aligned with one previous study using a nationally representative sample of adult women who had 33.3% of depressive symptoms assessed by PHQ-9, with younger women aged 18–38 experiencing depression (38.8%) during the pandemic in the U.S [45]. Notably, we observed that reproductive-aged women who self-identified as Other or Mixed race/ethnicity experienced the highest prevalence of depression and/or anxiety symptoms. This result aligns with a prior study examining the prevalence of depression in a nationally representative sample of U.S. adults (52% women), which found that participants identifying as Other or Mixed race/ethnicity had the highest prevalence of depressive symptoms as measured by PHQ-9 during the pandemic [45]. Our research expanded the existing literature by demonstrating the prevalence of both depressive and anxiety symptoms in a sample of women of reproductive age during the COVID-19 pandemic. In addition, our results provide compelling evidence that identifying as Other or Mixed race/ethnicity is a significant risk factor for mental health challenges among reproductive-aged women in the U.S. This heightened vulnerability may be attributed to the complex interplay of social, political, and cultural traumas these women often face, including issues of identity, discrimination, and societal marginalization [46]. These findings underscore the critical importance of recognizing women of Other or Mixed race/ethnicity as a distinct and underserved population facing disproportionate mental health burdens.

Our study revealed that women who experienced more frequent discrimination reported greater depression and anxiety symptoms. These findings align with prior studies showing that racial/ethnic discrimination was positively associated with both depression and/or anxiety symptoms among reproductive-aged Hispanic [47–49], and Black/African American women [50–52] before the pandemic. Our findings extend this evidence, demonstrating that perceived discrimination attributed to various reasons (including gender, race, age, religion, weight, height, sexual orientation, education, or income) is a risk factor for mental health problems in reproductive-aged women during the COVID-19 pandemic. These results underscore the need for healthcare providers to adopt a holistic approach to mental health care in this population. Clinicians should conduct comprehensive assessments that screen for both mental health symptoms and social stressors, particularly experiences of discrimination. Treatment plans should be individualized to address each woman’s unique circumstances, such as developing coping strategies for the particular forms of discrimination she encounters. This approach necessitates collaboration with mental health professionals and community organizations to address both clinical symptoms and underlying social determinants of health.

Our study provides preliminary evidence that decreased perceived social support may partially explain the association between discrimination and depression/anxiety symptoms among reproductive-aged women. Due to the cross-sectional design, causal inference cannot be established. However, the observed patterns suggest that women who experienced discrimination were more likely to perceive less available social support, which was associated with increased depression/anxiety symptoms. These findings contribute to the limited literature on social support as a potential mediator for the association between discrimination and mental health problems in reproductive-aged women in the U.S. Notably, these associations were consistent across NH-White, Hispanic, NH-Black/African American, and NH-Asian groups, but not among women identifying as the Mixed/Other race/ethnicity. Our results align with cross-sectional studies conducted in different populations outside the U.S. For example, among primarily Caucasian women with prior mental health diagnoses in Canada, social support mediated the association between stigma and emotional well-being [53]. Similarly, social support mediated the relationship between discrimination and emotional well-being among South Korean adults (47% women) in Japan [54]. Longitudinal studies are needed to establish the temporal precedence and directionality of the relationships among discrimination, social support, and mental health problems.

Our findings have important clinical and policy implications. The results suggest that increased social support may be associated with reduced effects of discrimination on mental health. Therefore, healthcare providers should adopt an integrated approach that addresses both mental health screening and the social determinants of health. Specifically, clinicians should routinely screen for depression and anxiety symptoms, assess whether women have experienced discrimination, and actively connect them with social support resources when needed. Additional evidence-based strategies to implement this approach include: (1) empowering women to seek help, build, and maintain supportive relationships; (2) connecting women with support groups, community organizations, or social services that can offer additional resources as needed; and (3) advocating for policies and practices that combat diverse forms of discrimination and promote inclusive environments for women in various settings. By addressing both mental health symptoms and their social determinants, healthcare systems can more effectively support the emotional well-being of reproductive-aged women experiencing discrimination.

### Strengths and limitations

A major strength of this study is that, to our knowledge, it is the first to examine how social support is associated with the relationship between discrimination and depression/anxiety symptoms among women in the U.S. Study findings were further strengthened by the fact, we focused on women aged 18–50, a demographic identified as having increased susceptibility to mental illness during the COVID-19 pandemic [5]. In addition, all data in our study were collected during the COVID-19 pandemic, which allowed us to elucidate the contextual effects of lockdown, fears of illness, economic constraints, and/or social isolation on discrimination and mental health. Furthermore, we investigated both depressive and anxiety symptoms, providing a more comprehensive view of mental health outcomes.

A few noteworthy limitations are warranted. First, given the cross-sectional nature of our data, our findings should be interpreted as exploratory associations rather than evidence of causal mediation. Alternative explanations, including reverse causation (whereby high depression/anxiety symptoms reduce social support or increase perceived discrimination) and bidirectional relationships, cannot be ruled out. Second, depression/anxiety symptoms were collected through questionnaires rather than diagnoses of mental health problems by qualified clinicians, potentially introducing self-reporting bias. Third, U.S. public health measures were implemented regionally, potentially resulting in differential mental health experiences across time and space. Additionally, other traumatic events occurred during this period, further complicating the mental health landscape. Notably, the killing of George Floyd in police custody on May 25, 2020, and subsequent widespread protests against racial injustice may have heightened experiences of discrimination and mental health distress, particularly among racial/ethnic minorities. Our analyses focused on reproductive-aged women who completed their first COPE survey between May and July 2020, and these temporal, regional, and sociopolitical factors might influence outcome assessments. Fourth, Among reproductive-aged women in version 8 of the AoURP, the majority were highly educated (72% held a bachelor's degree or higher) and identified as non-Hispanic White (73%). This sample composition may introduce a selection bias to our results and limit the generalization of our findings. However, it’s noteworthy that AoURP actively extended community engagement efforts, intentionally reaching out and recruiting individuals from marginalized communities. Future studies could replicate and extend these findings by analyzing additional waves of the COPE survey from the AoURP dataset or by designing a longitudinal study that will recruit participants from more diverse ethnic, socioeconomic, and regional backgrounds, thereby strengthening the generalizability of the results.

## Conclusions

Our study reveals that approximately one-third of reproductive-aged women participating in the AoURP experienced moderate-to-severe depressive/anxiety symptoms during the early COVID-19 pandemic in the U.S. We contribute new insights to the field by identifying associations among discrimination, social support, and depression/anxiety symptoms among these women. These findings underscore the potential importance of social support for emotional well-being, particularly among women of reproductive age in the U.S. Importantly, our results suggest that strengthening the availability and accessibility of social support resources may be a promising strategy to address mental health concerns related to experiences of discrimination. Healthcare providers and policymakers should prioritize interventions that combat various forms of discrimination, empower women to cultivate supportive social networks, facilitate access to community resources, and foster inclusive environments.

## Data Availability

This study used data from the *All of Us* Research Program’s [Registered] Tier Dataset [version 8], available to authorized users on the [Researcher Workbench](https://workbench.researchallofus.org).

## Abbreviations

COVID-19: Coronavirus disease
U.S.: United States
AoURP: All of Us Research Program
NIH: National Institutes of Health
EHRs: Electronic Health Records
COPE: COVID-19 Participant Experience
PHQ-9: Patient Health Questionnaire-9
GAD-7: Generalized Anxiety Disorder-7
NH: Non-Hispanic
GED: General Educational Development

## References

[1] The Lancet Psychiatry. COVID-19 and mental health. Lancet Psychiatry. 2021;8:87. 10.1016/S2215-0366(21)00005-5. 33485416 10.1016/S2215-0366(21)00005-5 PMC7825966

[2] Yeh JC, Subbiah IM, Dhawan N, Thompson BW, Hildner Z, Jawed A, et al. Visitation policies at NCI-designated comprehensive cancer centers during the COVID-19 pandemic. SUPPORT CARE CANCER. 2021;29:4895–8. 10.1007/s00520-021-06183-z. 33796936 10.1007/s00520-021-06183-z PMC8016614

[3] Zhou X, Nguyen-Feng VN, Wamser-Nanney R, Lotzin A. Racism, posttraumatic stress symptoms, and racial disparity in the U.S. COVID-19 syndemic. Behav Med. 2022;48:85–94. 10.1080/08964289.2021.2006131. 35318897 10.1080/08964289.2021.2006131

[4] McClure ES, Vasudevan P, Bailey Z, Patel S, Robinson WR. Racial capitalism within public health-how occupational settings drive COVID-19 disparities. Am J Epidemiol. 2020;189:1244–53. 10.1093/aje/kwaa126. 32619007 10.1093/aje/kwaa126 PMC7337680

[5] Almeida M, Shrestha AD, Stojanac D, Miller LJ. The impact of the COVID-19 pandemic on women’s mental health. Arch Womens Ment Health. 2020;23:741–8. 10.1007/s00737-020-01092-2. 33263142 10.1007/s00737-020-01092-2 PMC7707813

[6] Hermann A, Deligiannidis KM, Bergink V, Monk C, Fitelson EM, Robakis TK, et al. Response to SARS-Covid-19-related visitor restrictions on labor and delivery wards in New York City. Arch Womens Ment Health. 2020;23:793–4. 10.1007/s00737-020-01030-2. 32296947 10.1007/s00737-020-01030-2 PMC7156902

[7] Nations U. Make the prevention and redress of violence against women a key part of national response plans for COVID-19. United Nations. https://www.un.org/en/un-coronavirus-communications-team/make-prevention-and-redress-violence-against-women-key-part. Accessed 18 Feb 2024.

[8] Street AE, Dardis CM. Using a social construction of gender lens to understand gender differences in posttraumatic stress disorder. Clin Psychol Rev. 2018;66:97–105. 10.1016/j.cpr.2018.03.001. 29580673 10.1016/j.cpr.2018.03.001

[9] Mouzon DM, Taylor RJ, Woodward A, Chatters LM. Everyday racial discrimination, everyday non-racial discrimination, and physical health among African Americans. J Ethn Cult Divers Soc Work. 2017;26:68–80. 10.1080/15313204.2016.1187103. 28286428 10.1080/15313204.2016.1187103 PMC5342249

[10] Williams DR, Haile R, Mohammed SA, Herman A, Sonnega J, Jackson JS, et al. Perceived discrimination and psychological well-being in the U.S.A. and South Africa. Ethn Health. 2012;17:111–33. 10.1080/13557858.2012.654770. 22339224 10.1080/13557858.2012.654770 PMC3468317

[11] Berger M, Sarnyai Z. More than skin deep: stress neurobiology and mental health consequences of racial discrimination. Stress. 2015;18:1–10. 10.3109/10253890.2014.989204. 25407297 10.3109/10253890.2014.989204

[12] Wang ML, Narcisse M-R. Discrimination, depression, and anxiety among U.S. adults. JAMA Netw Open. 2025;8:e252404. 10.1001/jamanetworkopen.2025.2404. 40152858 10.1001/jamanetworkopen.2025.2404 PMC11953758

[13] Bailey ZD, Moon JR. Racism and the political economy of COVID-19: will we continue to resurrect the past? J Health Polit Policy Law. 2020;45:937–50. 10.1215/03616878-8641481. 32464657 10.1215/03616878-8641481

[14] Egede LE, Walker RJ. Structural racism, social risk factors, and COVID-19 — a dangerous convergence for Black Americans. N Engl J Med. 2020;383:e77. 10.1056/NEJMp2023616. 32706952 10.1056/NEJMp2023616 PMC7747672

[15] Lee YH, Liu Z, Fatori D, Bauermeister JR, Luh RA, Clark CR, et al. Association of everyday discrimination with depressive symptoms and suicidal ideation during the COVID-19 pandemic in the all of us research program. JAMA Psychiatry. 2022;79:898–906. 10.1001/jamapsychiatry.2022.1973. 35895053 10.1001/jamapsychiatry.2022.1973 PMC9330278

[16] Liu Y, Finch BK, Brenneke SG, Thomas K, Le PD. Perceived discrimination and mental distress amid the COVID-19 pandemic: evidence from the understanding America study. Am J Prev Med. 2020;59:481–92. 10.1016/j.amepre.2020.06.007. 32829968 10.1016/j.amepre.2020.06.007 PMC7336127

[17] Choi KW, Lee YH, Liu Z, Fatori D, Bauermeister JR, Luh RA, et al. Social support and depression during a global crisis. Nat Mental Health. 2023;1:428–35. 10.1038/s44220-023-00078-0.

[18] Kawachi I, Berkman LF. Social ties and mental health. J Urban Health. 2001;78:458–67. 10.1093/jurban/78.3.458. 11564849 10.1093/jurban/78.3.458 PMC3455910

[19] Zhang X, Xiao Y, Xu P, Dong S. Social support and mental health symptoms during the COVID-19 pandemic: A comprehensive meta-analysis unveils limited protective effects. J Pac Rim Psychol. 2025;19:18344909251324571. 10.1177/18344909251324571.

[20] Barrera M. Jr. Models of social support and life stress: Beyond the buffering hypothesis. In: Cohen LH, editor. Life events and psychological functioning: Theoretical and methodological issues. (pp. 211–36). Newbury Park (CA): Sage; 1988.

[21] Prelow HM, Mosher CE, Bowman MA. Perceived racial discrimination, social support, and psychological adjustment among African American college students. J Black Psychol. 2006;32:442–54. 10.1177/0095798406292677.

[22] Vargas SM, Huey SJ, Miranda J. A critical review of current evidence on multiple types of discrimination and mental health. Am J Orthopsychiatry. 2020;90:374–90. 10.1037/ort0000441. 31999138 10.1037/ort0000441

[23] Qin W, Nguyen AW, Mouzon DM, Hamler TC, Wang F. Social support, everyday discrimination, and depressive symptoms among older African Americans: a longitudinal study. Innov Aging. 2020;4:igaa032. 10.1093/geroni/igaa032. 32995567 10.1093/geroni/igaa032 PMC7508349

[24] Brandt L, Liu S, Heim C, Heinz A. The effects of social isolation stress and discrimination on mental health. Transl Psychiatry. 2022;12:1–11. 10.1038/s41398-022-02178-4. 36130935 10.1038/s41398-022-02178-4 PMC9490697

[25] Guntzviller LM, Williamson LD, Ratcliff CL. Stress, social support, and mental health among young adult hispanics. Fam Community Health. 2020;43:82–91. 10.1097/FCH.0000000000000224. 31764309 10.1097/FCH.0000000000000224

[26] Steers M-LN, Chen T-A, Neisler J, Obasi EM, McNeill LH, Reitzel LR. The buffering effect of social support on the relationship between discrimination and psychological distress among church-going African-American adults. Behav Res Ther. 2019;115:121–8. 10.1016/j.brat.2018.10.008. 30415761 10.1016/j.brat.2018.10.008 PMC6409102

[27] Alvarez D, Adynski H, Harris R, Zou B, Taylor JY, SantosJr HP. Social Support Is Protective Against the Effects of Discrimination on Parental Mental Health Outcomes. J Am Psychiatr Nurses Assoc. 2024;30:953–65. 10.1177/10783903241243092. 38600825 10.1177/10783903241243092 PMC11558929

[28] Held ML, First JM, Huslage M. Effects of COVID-19, discrimination, and social support on Latinx adult mental health. J Immigr Minor Health. 2022;24:1446–58. 10.1007/s10903-022-01382-0. 35841445 10.1007/s10903-022-01382-0 PMC9288212

[29] Aviles Gonzalez CI, Barrui V, Migliaccio GM, Curcio F, Gioiello G, Romero Z, et al. Gender differences in the perceived impact of major depressive disorder on quality of life: A cross-sectional population study. J Clin Med. 2025;14:5984. 10.3390/jcm14175984. 40943744 10.3390/jcm14175984 PMC12429698

[30] Farhane-Medina NZ, Luque B, Tabernero C, Castillo-Mayén R. Factors associated with gender and sex differences in anxiety prevalence and comorbidity: A systematic review. Sci Prog. 2022;105:00368504221135469. 10.1177/00368504221135469. 36373774 10.1177/00368504221135469 PMC10450496

[31] Arilha M, Carvalho AP, Forster TA, Rodrigues CVM, Briguglio B, Serruya SJ. Women’s mental health and COVID-19: increased vulnerability and inequalities. Front Glob Women’s Health. 2024. 10.3389/fgwh.2024.1414355. 5. 10.3389/fgwh.2024.1414355 PMC11480059 39416672

[32] Lassi ZS, Wade JM, Ameyaw EK. Stages and future of women’s health: A call for a life-course approach. Womens Health (Lond). 2025;21:17455057251331721. 10.1177/17455057251331721. 40258196 10.1177/17455057251331721 PMC12035259

[33] Xu H, Ren J, Liu H, Xu Q, Yuan R, Zhuang D et al. Global, regional, and national burden and trends of mental disorders in women of childbearing age: a systematic analysis based on the global burden of disease study 2021. Ann Med. 57:2576642. 10.1080/07853890.2025.2576642. 10.1080/07853890.2025.2576642 PMC12590574 41190594

[34] Data snapshots –. All of Us research hub. https://www.researchallofus.org/data-tools/data-snapshots/. Accessed 18 Aug 2024.

[35] Crawford S, Smith RA, Kuwabara SA, Grigorescu V. Risks factors and treatment use related to infertility and impaired fecundity among reproductive-aged women. J Womens Health (Larchmt). 2017;26:500–10. 10.1089/jwh.2016.6052. 28186831 10.1089/jwh.2016.6052 PMC5576020

[36] Sadecki E, Weaver A, Zhao Y, Stewart EA, Ainsworth AJ. Fertility trends and comparisons in a historical cohort of US women with primary infertility. Reprod Health. 2022;19:13. 10.1186/s12978-021-01313-6. 35042514 10.1186/s12978-021-01313-6 PMC8764822

[37] Williams DR, Yu Y, Jackson JS, Anderson NB. Racial differences in physical and mental health: Socio-economic status, stress and discrimination. J Health Psychol. 1997;2:335–51. 10.1177/135910539700200305. 22013026 10.1177/135910539700200305

[38] Sherbourne CD, Stewart AL. The MOS social support survey. Soc Sci Med. 1991;32:705–14. 10.1016/0277-9536(91)90150-B. 2035047 10.1016/0277-9536(91)90150-b

[39] Kroenke K, Spitzer RL, Williams JBW. The PHQ-9: Validity of a brief depression severity measure. J Gen Intern Med. 2001;16:606–13. 10.1046/j.1525-1497.2001.016009606.x. 11556941 10.1046/j.1525-1497.2001.016009606.x PMC1495268

[40] Spitzer RL, Kroenke K, Williams JBW, Löwe B. Generalized anxiety disorder 7. 2011. 10.1037/t02591-000.

[41] Dong Y, Peng C-YJ. Principled missing data methods for researchers. Springerplus. 2013;2:222. 10.1186/2193-1801-2-222. 23853744 10.1186/2193-1801-2-222 PMC3701793

[42] Schafer JL. Multiple imputation: a primer. Stat Methods Med Res. 1999;8:3–15. 10.1177/096228029900800102. 10347857 10.1177/096228029900800102

[43] Boyd RW, Lindo EG, Weeks LD, McLemore MR. On racism: a new standard for publishing on racial health inequities. Health Affairs Forefr. 10.1377/forefront.20200630.939347.

[44] Tingley D, Yamamoto T, Hirose K, Keele L, Imai K. Mediation: R package for causal mediation analysis. UCLA Statistics/American Statistical Association; 2014.

[45] Ettman CK, Abdalla SM, Cohen GH, Sampson L, Vivier PM, Galea S. Prevalence of depression symptoms in U.S. adults before and during the COVID-19 pandemic. JAMA Netw Open. 2020;3:e2019686. 10.1001/jamanetworkopen.2020.19686. 32876685 10.1001/jamanetworkopen.2020.19686 PMC7489837

[46] Tabb KM. Racial categorization in women’s mental health research fails to meet the needs of multiracial, biracial, and mixed-race women in the United States. J Obstet Gynecol Neonatal Nurs. 2025;54:5–8. 10.1016/j.jogn.2024.12.002. 39709182 10.1016/j.jogn.2024.12.002

[47] Mata-Greve F, Torres L. Ethnic discrimination, sexism, and depression among Latinx women: The roles of anxiety sensitivity and expressive suppression. J Latinx Psychol. 2020;8:317–31. 10.1037/lat0000154.

[48] Santos HP, Adynski H, Harris R, Bhattacharya A, Incollingo Rodriguez AC, Cali R, et al. Biopsychosocial correlates of psychological distress in Latina mothers. J Affect Disord. 2021;282:617–26. 10.1016/j.jad.2020.12.193. 33445084 10.1016/j.jad.2020.12.193 PMC7889736

[49] Sluiter F, Incollingo Rodriguez AC, Nephew BC, Cali R, Murgatroyd C, Santos HP. Pregnancy associated epigenetic markers of inflammation predict depression and anxiety symptoms in response to discrimination. Neurobiol Stress. 2020;13:100273. 10.1016/j.ynstr.2020.100273. 33344726 10.1016/j.ynstr.2020.100273 PMC7739167

[50] Giurgescu C, Zenk SN, Engeland CG, Garfield L, Templin TN. Racial discrimination and psychological wellbeing of pregnant women. MCN: Am J Maternal/Child Nurs. 2017;42:8. 10.1097/NMC.0000000000000297. 10.1097/NMC.0000000000000297 PMC5143203 27749288

[51] Louis CC, Buchanan NT, Moser JS. Associations between experiences of discrimination, anxiety, and mood symptoms in Black women: Investigating the mediating role of attentional control. J Mood Anxiety Disord. 2024;7:100070. 10.1016/j.xjmad.2024.100070. 40656832 10.1016/j.xjmad.2024.100070 PMC12244055

[52] Perry BL, Harp KLH, Oser CB. Racial and gender discrimination in the stress process: implications for African American women’s health and well-being. Sociol Perspect. 2013;56:25–48. 24077024 PMC3783344

[53] Kondrat DC, Link to external site this link will open in a new tab, Sullivan WP, Wilkins B, Barrett BJ, Beerbower E. The mediating effect of social support on the relationship between the impact of experienced stigma and mental health. Stigma and Health. 2018;3:305–14. 10.1037/sah0000103.

[54] Park J, Joshanloo M. Mediating and moderating effects of perceived social support on the relationship between discrimination and well-being: A study of South Koreans living in Japan. Front Psychol. 2022;13:922201. 10.3389/fpsyg.2022.922201. 35967623 10.3389/fpsyg.2022.922201 PMC9366095

